# *A Hospital and Health Service v C* [2025] QSC 178—Termination of Pregnancy for a Minor: Consideration of “Best Interests” Post Enactment of the *Human Rights Act 2019* (Qld)

**DOI:** 10.1007/s11673-025-10515-7

**Published:** 2025-10-27

**Authors:** Michaela Estelle Okninski

**Affiliations:** Adelaide Law School, Faculty of Arts, Business, Law and Economics, The University of Adelaide, Adelaide, Australia

**Keywords:** Gillick competence, parens patriae jurisdiction, best interests of a child, termination of pregnancy, human rights

## Introduction

Judicial determination of whether medical treatment is in a child’s best interests usually raises challenging legal and ethical issues. *A Hospital and Health Service v C* [2025] QSC 178 (‘*AHHS v C*’) exemplifies this assertion, where the Supreme Court of Queensland was called upon to consider whether a termination of pregnancy was in the best interests of an eleven-year-old child.

At law, consent to medical treatment can be provided by a minor if they are *Gillick* competent, or by a parent or legal guardian if *Gillick* competence cannot be demonstrated (see *Gillick v West Norfolk and Wisbech Area Health* Facility [1986] AC 112; *Secretary Department of Health and Community Services v JWB and SMB* (1992) 175 CLR 218 (“*Marions Case*”)). However, as recognized by the High Court of Australia in *Marion’s Case*, some medical treatments—special medical procedures—are beyond the scope of parental authority and require court authorization to proceed. Termination of pregnancy for a minor falls within the category of special medical procedures (*State of Queensland v B* [2008] 2 Qd R 562, at [17]), and such cases require the court to act as “supreme parent of children” and determine what is in a child’s best interests [*State of Queensland v Nolan* [2002] 1 Qd R 454, 455 at [7], cited by Williams J in *A Hospital and Health Service v C* [2025] QSC 178 at [38]].

The present case, brought before the Supreme Court of Queensland’s (QSC) parens patriae jurisdiction, required Williams J to consider whether the Respondent, C, was G*illick* competent to consent to a termination of pregnancy, and if not, whether a termination of pregnancy was in her best interests. While similar cases have previously been brought before the QSC for determination (see *State of Queensland v B* [2008] 2 Qd R 562; *Central Queensland Hospital and Health Services v Q* [2017] 1 Qd R 87), *AHHS v C* is of interest as this case considers new issues, namely the interaction between the QSC’s parents patriae jurisdiction and the *Human Rights Act *2019 (Qld), which commenced operation on January 1, 2020.

### Background

In *AHHS v C*, the Applicant, a hospital, sought orders authorizing the Applicant’s servants and agents to perform a termination of pregnancy on the Respondent, C. At the time of the hearing, C was eleven years old and eight weeks pregnant. Given the sensitive nature of the case, protecting C’s identity was of paramount importance and deemed critical to her welfare, and as such, Williams J avoided providing a detailed factual background (at [16]). Furthermore, consistent with the Court’s overarching obligation to act in the child’s best interests, the Court deemed it necessary to make additional orders to safeguard C’s identity, including suppressing and restricting the court file, the transcript, reasons for decision, and suppressing the names of relevant parties including C’s mother, C’s treating medical team, and the Applicant hospital ([72]-[75]). Therefore, little is known about C’s background or personal circumstances. However, it was noted that C’s mother was her litigation guardian.

To assist the Court in determining whether a termination of pregnancy was in C’s best interests, King’s Counsel was appointed amicus curiae, and Counsel and Crown Law, on behalf of the Attorney-General, were appointed amici curiae to assist with the human rights considerations that arose under the *Human Rights Act *2019 (Qld).

### Issues

The paramount issues before Williams J for consideration were:

1. Whether C was *Gillick* competent to consent to medical/surgical termination of pregnancy, and if not,
2. Whether it was in C’s best interests to have a medical/surgical termination of pregnancy.

It is necessary to note that the Applicant also sought authorization to provide additional medical treatment to the Respondent by implanting a contraceptive device. However, determination of this issue was adjourned due to the additional factual and legal issues raised and the termination of pregnancy being time critical [[1]–[4]].

### Findings and Outcome

#### Gillick Competence

In addressing the threshold issue of whether C was competent to consent to the medical treatment proposed, Williams J stated that *Gillick* competence would be satisfied if a minor has “achieved a sufficient understanding and intelligence to enable [them] to understand fully what is proposed ([12]),” noting that age, while a relevant consideration, was not determinative ([23]). Her Honour continued, observing that assessment of understanding requires consideration of:

- The child’s insight into their condition;
- Their understanding of the nature of the treatment and its effects;
- Their understanding of the consequences and potential side effects of the treatment; and
- Their ability to comprehend and retain information ([24]).

In relation to understanding the nature, effects, consequences and potential side effects of a termination of pregnancy, Williams J was guided by two previous decisions of the QSC, both concerning a termination of pregnancy for a minor—*State of Queensland v B* [2008] 2 Qd R 562 (“*SQ v B*”) and *Central Queensland Hospital and Health Services v Q* [2017] 1 Qd R 87 (“*CQHHS v Q*”).

*SQ v B* – a case concerning a twelve-year-old child who was eighteen weeks pregnant—Wilson J opined that “it seems unlikely that a 12 year old child of average intelligence and maturity could fully understand the significance of a termination of pregnancy, including the immediate and long term risks to herself as the mother of the baby [16].” As the Respondent, B, was of less than average intelligence and maturity, it was found that they were not capable of fully appreciating these risks. This was further explored in the later case of *CQHHS v Q*.

In *CQHHS v Q*, it was noted that the Respondent, Q, could demonstrate a very good understanding of the risks associated with a termination of pregnancy, and as such, more weight could be given to her views. However, McMeekin J found that Q’s ability to make an informed decision could not be established as this required understanding of the long term consequences of a decision *not to terminate* (McMeekin J at [30], cited in *A Hospital and Health Service v C* [2025] QSC 178 at [20]). Regarding the long term consequences of continuing a pregnancy, the Court accepted that, “[Q] had little or no idea about the process of pregnancy and had no idea of the realistic emotional and physical demands that would be part of caring for and raising a child (at [31]).” Expanding on this point, McMeekin J emphasized that “very few 12 year olds could have the maturity to comprehend the impact a decision [not to terminate] might have on them in the longer term (*CQHHS v Q* at [32]).” Thus, these two cases suggest that *Gillick* competence requires not only sufficient maturity to comprehend the nature, effects, consequences, and potential side effects of terminating a pregnancy itself, but also sufficient maturity to understand the long-term consequences of continuing a pregnancy and parenthood itself, which is unlikely to satisfied in younger children.

In applying these considerations to the facts of C, Williams J found that C had not “achieved a sufficient understanding and intelligence to enable her to understand fully what is proposed regarding her options (at [37]).” Central to this finding was that during various appointments with the treating adolescent psychiatrist and treating paediatrician, C demonstrated behaviours indicating that she was “emotionally and psychologically immature” (at [28]–[30]). Furthermore, it was found that while C could demonstrate basic understanding of the concept of being pregnant by stating that she has a “baby in her tummy”, and that a termination of pregnancy would mean that she is “not pregnant” ([25]), she had only a minimal understanding of the consequences and long term risks not only associated with a termination of pregnancy, but also concerning continuing a pregnancy ([25]-[27]). It was also highlighted that C’s understanding of a termination of pregnancy appeared to be transient, and she was “unable to recall what had previously been discussed regarding … the termination procedures (at [28]).” Thus, Williams J concluded that C was not *Gillick* competent to consent to a termination of pregnancy and proceeded to consider whether a termination of pregnancy was in C’s best interests.

## Was the Supreme Court’s Parens Patriae Jurisdiction Invoked?

Given that C was not *Gillick* competent, and that consent to a termination of pregnancy for a minor is beyond the scope of parental authority, Williams J determined that the QSC’s parens patriae jurisdiction was enlivened ([38]). However, Her Honour observed that it was currently unsettled whether there was a direct application of human rights under the *Human Rights Act *2019 (Qld) (“*HRA*”*)* (at [41]-[42]]) Williams J left this question unresolved and instead directly addressed what human rights *may* be relevant to informing the Court when exercising its parens patriae jurisdiction ([39]–[42]).

### Interaction With the Human Rights Act 2019 (Qld)

Prior to addressing the specific human rights engaged under the *HRA*, Williams J first considered whether the Court’s parens patriae jurisdiction also extends to the unborn child ([45]). This was a necessary preliminary issue to resolve because if the parens patriae jurisdiction extended to C’s unborn child, so too would human rights considerations under the *HRA*, which would then need to be weighed and balanced against C’s human rights.

Her Honour noted that at common law, the parens patriae jurisdiction only applies to the minor whose interests the court has been called upon to consider and not the unborn child (at [45], referring to *K v T* [1983] 1 Qd R 396). However, whether the common law rule was displaced by the *HRA* required further consideration. In addressing this point, Williams J turned to Section 106 of the *HRA* which provides that “nothing in the Act affects any law relating to termination of pregnancy (at [45]),” thus, necessitating discussion of the law relating to a termination of pregnancy.

In Queensland, a termination of pregnancy is governed by the *Termination of Pregnancy Act *2018 (Qld) (“*TPA*”). Section 5 of the *TPA* authorizes a medical practitioner to perform a termination of pregnancy on a woman who is not more than twenty-two weeks pregnant. However, as the *TPA* is silent concerning the specific requirements of consent to a termination of pregnancy, reference to the Explanatory Note accompanying the Termination of Pregnancy Bill 2018 (Qld) was required to ascertain parliamentary intention.

Justice Williams stated that when introducing the Termination of Pregnancy Bill 2018 (Qld), Parliament did not intend to replace the common law principles governing consent to a termination of pregnancy for a minor. In quoting the Explanatory Note, it was stated that “an order by the Supreme Court in relation to a termination [of pregnancy for a minor] is required. In making such an order, *the court must act in the best interests of the pregnant child*. Treatment in the absence of consent may give rise to civil or criminal liability” ([48], emphasis added).” Therefore, as the law only requires a Court to consider the best interests of the pregnant child, and Section 106 of the *HRA* does not affect any law relating to a termination of pregnancy, the Court’s parens patriae jurisdiction did not extend to the unborn child.

### What Human Rights Were Engaged?

Justice Williams, assisted by Counsel and Crown Law for the Attorney-General as amici curiae, identified several human rights under the *HRA* applicable to determining whether a termination of pregnancy was in C’s best interests ([51]). Central to this inquiry was that “every child has the right, without discrimination, to the protection that is needed by the child, and is in the child’s best interests, because of being a child (*HRA* s 26(2), at [50])”. It was further accepted that:

- the right to recognition and equality before the law;
- the right to life;
- the right to be free from medical treatment without full free and informed consent;
- the right not to have privacy interfered with unlawfully or arbitrarily, noting that the human rights concept of privacy extends to mental and bodily integrity;
- the right to security of person; and
- the right of access to health services without discrimination, which includes access to sexual and reproductive health services (at [51]),

were also engaged,^1^ noting that consideration of these rights was a balancing exercise where competing considerations arose ([53]-[54]). It was also stated that consideration of human rights involved taking into account C’s stated views and preferences ([57]). It is necessary to highlight that although multiple provisions of the *HRA* were identified, Williams J primarily focused on C’s right to privacy, which encompasses the right to mental and bodily integrity.

It was observed that while a termination of pregnancy would “involve some level of pain and distress ([53])”, and thus, interfere with C’s mental and bodily integrity, it was found that continuing the pregnancy would have a greater impact on her mental and bodily integrity ([53]-[62]). Central to this finding was that there was “overwhelming medical evidence [indicating] that the risks involved in continuing C’s pregnancy [were] higher than the risks involved in termination ([54]).” Concerning the risks to health, it was accepted that due to her young age, C was at a higher risk of “hypertensive disorders of pregnancy … more often complicated by pre-term birth and low birth weight infants [[61]].” Furthermore, it was found that C had a “substantial risk for both mental and psychosocial health and wellbeing if the pregnancy continue[d] ([61]),” which was supported by the treating adolescent psychiatrist who opined that C was “at a much higher risk of mental ill health” due to her particular circumstances ([62]).

In noting that human rights and best interests considerations involve taking into account the expressed views and wishes of the individual whose rights are impacted ([57]), Williams J highlighted that C had expressed on more than one occasion her view to not continue with the pregnancy, which was also supported by her mother ([57]).

### Outcome

In finding that C was not *Gillick* competent to consent to a termination of pregnancy, and that it was in her best interests to undergo a termination, the Court made an order permitting C to undergo a termination of pregnancy, and permitting the Applicant, including its servants and agents, to perform a medical or surgical termination of pregnancy, including any associated medical procedure, on the Respondent ([63]-[66] and [69]-[71]).

## Conclusion

*AHHS v C* is an interesting case as it considers challenging legal, ethical, and moral issues relating to a termination of pregnancy for a minor against the backdrop of recently enacted human rights legislation. This decision is important as it clarifies that in cases concerning a termination of pregnancy for a minor, the court’s parens patriae jurisdiction is not altered by the *Human Rights Act *2019 (Qld). Thus, the paramount consideration is what is in the pregnant child’s best interests and this does not extend to the unborn baby. Additionally, this case provides guidance for future analogous cases concerning what human rights principles may be engaged and how to balance competing considerations when determining whether a termination of pregnancy is in the best interests of a child.

## Data Availability

Not Applicable.

## Footnotes

1 See *Human Rights Act *2019 (Qld) ss 15, 16, 17(c), 25(a), 29(1), 37.

